# Road vehicle collision suicide in Australia: Trends, collision types, and individual characteristics

**DOI:** 10.1371/journal.pone.0299590

**Published:** 2024-04-30

**Authors:** Phillip C. F. Law, Lay San Too, Mathew J. Spittal, Jane Pirkis, Angela J. Clapperton

**Affiliations:** Centre for Mental Health, Melbourne School of Population and Global Health, The University of Melbourne, Parkville, Victoria, Australia; Al Mansour University College-Baghdad-Iraq, IRAQ

## Abstract

**Background:**

Suicide by road vehicle collision in Australia is under-explored with mixed findings. We aimed to address this research gap by examining time trends, different types of vehicle collision, and individual characteristics related to vehicle-collision suicide.

**Method:**

We retrospectively analyzed deaths by suicide between 1st January 2001 and 31st December 2017 in Australia, using coronial records from the National Coronial Information System. The travel mode used and collision counterpart were retrieved from records of death by vehicle-collision suicide using all available information. We conducted negative binomial regression analysis to examine annual changes in suicide rate by vehicle collision on a public road (N = 640) and other methods of suicide (N = 41,890), and logistic regression analysis to examine individual characteristics associated with the likelihood of dying by suicide via road vehicle collision.

**Results:**

Overall, the national suicide rate involving road vehicle collision significantly increased, while the rate by other methods significantly decreased. Drivers accounted for 61% of suicide events by vehicle collision, of which 72% were single-vehicle collisions (commonly involving a tree). For multiple-vehicle collision suicide events, 82% involved collision with a truck. Pedestrians accounted for more than one-third of suicide events, of which 58% involved collision with a truck and 23% involved collision with a car/van. Individuals who were male (odds ratio 1.15; 95% CI 0.88–1.50), aged <25 years old (odds ratio 5.27; 95% CI 3.05–9.10), non-Indigenous (odds ratio 3.36; 95% CI 1.71–6.62), and born overseas (odds ratio 1.40; 95% CI 1.10–1.79) were more likely to die by vehicle-collision suicide than by other methods of suicide.

**Conclusions:**

This study provides a better understanding of road vehicle collision suicide in Australia and informs future research directions on topic. Our findings can be used to inform suicide prevention initiatives to reduce vehicle-collision suicide deaths.

## Introduction

The decision to use road vehicle collision as a suicide method could be influenced by factors such as perception of pain, likelihood of fatal outcome, ready access to vehicles, likelihood of bystander intervention, and perception that the event will be ruled as unintentional (in order to secure life insurance policy payment for family and protect family against social stigmatisation; [1]. Suicide events involving road vehicle collision involve a greater risk of serious injury and death to bystanders than other suicide methods [2, 3]. For suicide events involving vehicles with a large weight disparity (e.g., car/van and truck), almost 30% result in injury of another person [4] and almost 4% result in death of another person [5].

Road vehicle collision as a suicide method involves either a driver intentionally colliding into an object (e.g., road infrastructure, a natural fixed object, another vehicle) or a pedestrian (i.e., person on foot) purposefully placing themselves in the collision path of an oncoming vehicle. Drivers who died by suicide are relatively more common [5–8], and research has largely focused on single-vehicle collisions [9]. In Australia, only two studies have examined suicide events involving road vehicle collision, using motor vehicle collision data in Queensland. One study estimated that single-vehicle collisions constituted 55% of driver deaths by suicide between 2011 and 2015 [10]. The finding is consistent with the other Australian study that estimated 58.6% of driver deaths by suicide between 1990 and 2007 involved colliding into a tree or power pole [11]. Estimates for multiple-vehicle collisions differed in the two studies, with reports that 27.5% [11] and 80% [10] of driver deaths by suicide involved a collision with an oncoming truck.

Pedestrians who died by suicide have done so by jumping off road infrastructure or, more commonly, by stepping into the path of an oncoming vehicle [12]. The topic of pedestrian deaths by suicide has received much less focus in research than driver deaths by suicide. The limited research that has been done on the topic suggests that pedestrian deaths by suicide typically involve colliding into an oncoming heavy vehicle such as a truck [6].

Drivers who died by suicide are more often men, young to middle aged, unemployed, and not married [10, 11, 13–15]. The majority of drivers who died by suicide in Australia also consumed alcohol prior to the collision [11], and experienced legal and financial problems more so than people who used other suicide methods. These risk factors for vehicle-collision suicide mirror those reported in other countries [1, 3, 5, 7, 14–18], and are also commonly reported for other suicide methods.

There is still much that is not understood about vehicle-collision suicide in Australia. In particular, studies have yet to examine the suicide rate involving road vehicle collision over time, the proportion of different types of vehicle collision, and individual characteristics of those who died by vehicle-collision suicide. The current study addresses these gaps by analysing detailed case information from the National Coronial Information System. First, we identified all deaths by suicide involving road vehicle collision that occurred over a 17 year period (2001−2017) in Australia, during which an increase in road vehicle use was reported [19–21], and compared the annual trend to that of other suicide methods. Second, we examined the proportions of different travel modes used (e.g., car/van, on foot) and their collision counterparts (i.e., the corresponding object that the deceased collided with, e.g., tree, truck). Third, we identified individual characteristics associated with the likelihood of dying by vehicle-collision suicide and compared them to those of other suicide methods.

## Materials and methods

### Study design and definitions

We conducted a retrospective analysis of coronial data on deaths by suicide involving road vehicle collision and other methods, using data from each Australia state and territory. Deaths by suicide involving road vehicle collision were suicide fatalities that are a result of vehicle collision on a public road, including those cases where the death was brought about by a collision between vehicles, pedestrians walking into traffic, and where a vehicle was the location of death after being driven off-road (e.g., a vehicle colliding into a roadside tree or driven off a cliff). Deaths by suicide involving motor vehicle exhaust gas did not involve a road vehicle collision and were therefore classified as ‘other methods’ in this study. The study was reviewed and approved by the University of Melbourne’s Human Research Ethics Committee (Reference Number: 2021-13478-15976-5).

### Data source

Data on deaths with their intent type classified as intentional self-harm were obtained from the National Coronial Information System (NCIS; for further information on how intent is coded in the NCIS see [22]). The NCIS is a national internet-based data storage and retrieval system of Australian and New Zealand coronial records. Each record typically has a full text police summary of circumstances, autopsy report, toxicology report, coroner’s findings report (where procedures have been performed and are available), as well as identifying information and coded demographic information (e.g., marital and employment status at the time of death). The NCIS is the best source of data for national studies about suicide. We selected the time period between 1 January 2001 and 31 December 2017 in each Australian state or territory because at the time of data extraction the NCIS contained the most complete data on deaths by suicide for these years. Records with no known year of death were excluded. For each record of death by vehicle-collision suicide, we coded the travel mode used, which included car/van, motorcycle, truck, bicycle, mobility scooter, and on foot (i.e., pedestrian deaths by suicide). We also coded the collision counterpart using all available information, which included fixed objects (e.g., tree, concrete barrier, cliff base, unoccupied heavy vehicle), car/van, motorcycle, truck, bus, train, and tram. Records of death by suicide involving other methods (e.g., motor vehicle exhaust gassing, hanging, poisoning, jumping from height, firearm, cutting) were pooled as the comparison group. Population data was obtained from the Australian Bureau of Statistics. All data were obtained between 15 September 2021 and 27 January 2023.

We retrieved the following variables for each record of death by suicide: sex (male, female), age (<25, 25–34, 35–44, 45–54, 55–64, 65+ years), marital status (married/de facto, never married, divorced/separated/widowed), employment status (employed, unemployed, not in labor force), Indigenous origin (Indigenous, non-Indigenous) and country of birth (Australia, overseas).

### Statistical analysis

We performed descriptive analyses to estimate the annual suicide rate involving road vehicle collision and other methods in each Australia state and territory. To examine changes in suicide rate over time, we conducted univariate negative binomial regression analyses stratified by suicide method (road vehicle collision vs. other suicide methods). Our outcome was the count of the number of suicides in each year and the predictor was the year (entered as an indicator variable with 2001 as the reference year). Our model included an offset term for population size. For interpretation, we report all coefficients on the exponential scale as incidence rate ratios (IRRs). We performed descriptive analysis to estimate the proportion of each travel mode used and their collision counterparts. To examine the effects of individual characteristics of those who died by vehicle-collision suicide on the odds of dying by vehicle-collision suicide, we conducted logistic regression analyses in two stages. In the first stage we undertook univariate analyses on age, sex, marital status, employment status, Indigenous origin, and country of birth. Significant variables (p<0.05) from these analyses were subsequently entered into a multivariate model. All coefficients were exponentiated to interpret these results as odds ratios (ORs). Descriptive analysis was conducted using R (version 4.0.2). Negative binomial regression analysis and logistic regression analysis were conducted using Stata SE (version 13.1).

## Results

### Trends in deaths by suicide involving road vehicle collision and other methods

**Fig 1:**
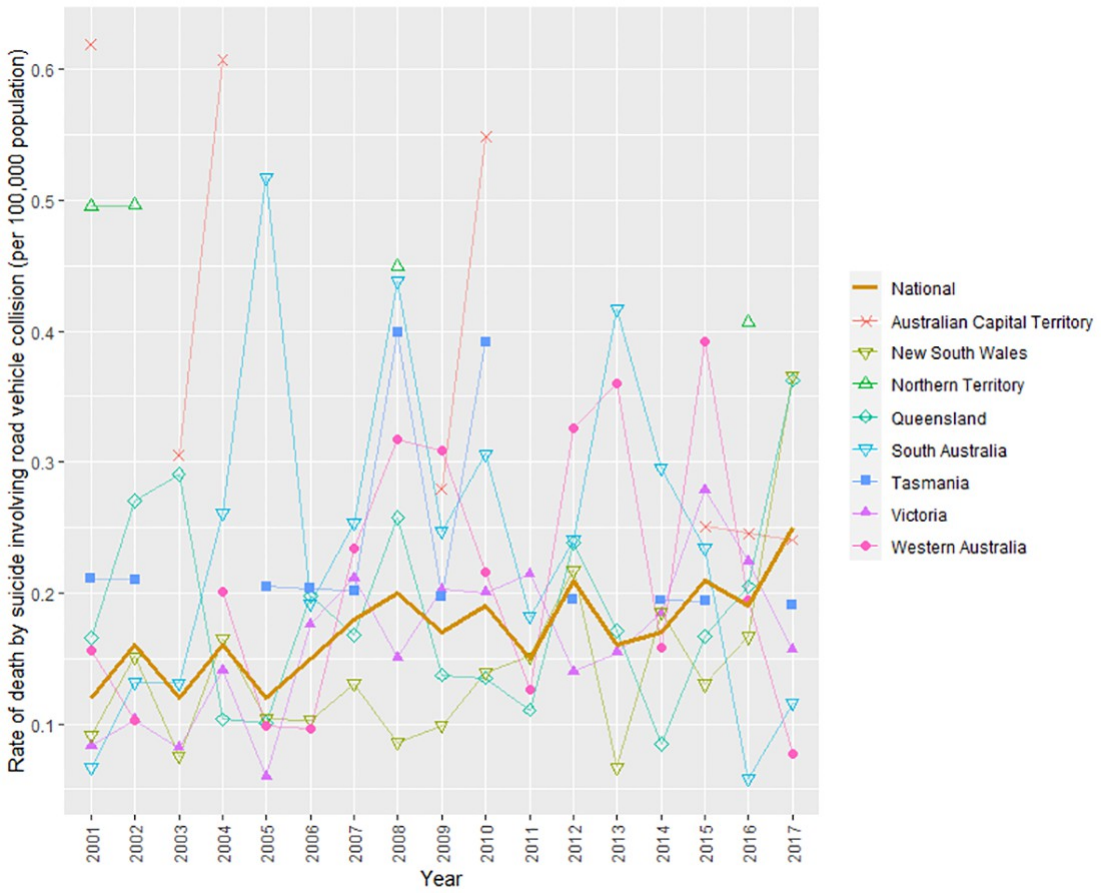
Suicide rates involving road vehicle collision between 2001 and 2017 in Australia.

**Fig 2:**
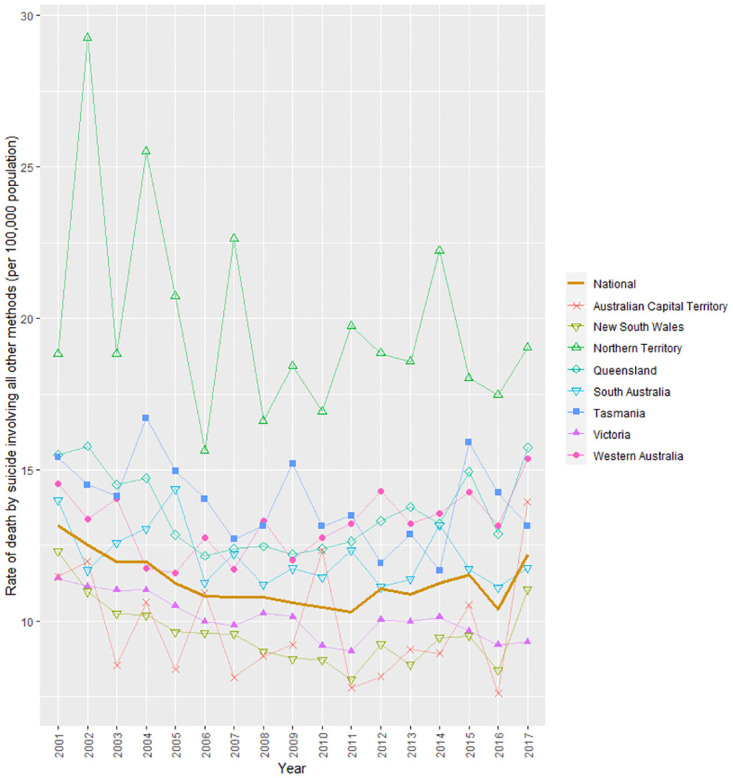
Suicide rates involving other methods between 2001 and 2017 in Australia.

A total of 640 individuals died by suicide in road vehicle collisions between 2001 and 2017. The travel mode used was obtained for 594 individuals and the collision counterpart was obtained for 573 individuals. Drivers accounted for 61% of people who died by vehicle-collision suicide and pedestrians who used an oncoming road vehicle for suicide accounted for the remaining 39%. Fig 1 shows an overall upward trend in the national suicide rate involving road vehicle collision over time (per 100,000 population). There was variability in the trends at the state/territory-level, with data from two states showing an increase in deaths by suicide involving road vehicle collision (Victoria and New South Wales) and the rest showing no change or a declining trend. A total of 41,890 individuals died by suicide involving other methods in the same time period. The series is characterised by an overall decline in the national suicide rate (Fig 2). State/territory-level variability in the trends was also observed, with three states showing a decline in deaths by suicide involving other methods (Victoria, New South Wales, and South Australia) and the remainder showing no change or an increasing trend.

Table 1 provides additional information about the increase in deaths by vehicle-collision suicide throughout the study period. It shows that the years that accounted for the increase were 2012 (IRR = 1.69), 2015 (IRR = 1.72), and 2017 (IRR = 2.05). In each of these years, the IRR was significantly higher than in 2001 (p≤0.001). In contrast, there was clear evidence of fewer deaths by suicide involving other methods in all years relative to the 2001 reference year.

**Table 1:** Incidence rate ratios for deaths by suicide involving road vehicle collision and other methods.

Year	Suicide by road vehicle collision			Suicide by other methods		
	IRR	95% CI	p-value	IRR	95% CI	p-value
2001^a^	1.00			1.00		
2002	1.28	0.75–2.18	0.37	0.95	0.90–1.00	0.07
2003	0.94	0.53–1.66	0.82	0.91	0.86–0.96	0.001
2004	1.29	0.76–2.19	0.35	0.91	0.86–0.96	0.001
2005	0.99	0.57–1.74	0.98	0.85	0.81–0.90	<0.001
2006	1.17	0.69–2.01	0.56	0.82	0.78–0.87	<0.001
2007	1.42	0.85–2.38	0.18	0.82	0.78–0.87	<0.001
2008	1.58	0.96–2.61	0.07	0.82	0.77–0.87	<0.001
2009	1.37	0.82–2.28	0.23	0.81	0.76–0.85	<0.001
2010	1.49	0.90–2.47	0.12	0.79	0.75–0.84	<0.001
2011	1.22	0.72–2.06	0.46	0.78	0.74–0.83	<0.001
2012	1.69	1.01–2.76	0.04	0.84	0.80–0.89	<0.001
2013	1.32	0.79–2.20	0.29	0.83	0.78–0.87	<0.001
2014	1.33	0.80–2.22	0.27	0.86	0.81–0.90	<0.001
2015	1.72	1.06–2.79	0.03	0.88	0.83–0.93	<0.001
2016	1.49	0.91–2.45	0.11	0.79	0.75–0.83	<0.001
2017	2.05	1.28–3.29	<0.01	0.93	0.88–0.98	<0.01

IRR, incidence rate ratio; CI, confidence interval.

^a^ Reference category.

### Types of road vehicle collisions

Of the drivers who died by suicide (N = 365), 264 (72%) were in single-vehicle collisions. For the remaining drivers who died by suicide (N = 101), 99 were in multiple-vehicle collisions of which 81 collided with an oncoming truck. Table 2 shows that when a car/van was driven as the travel mode for suicide, 74% of the collisions involved a fixed object (commonly a tree or concrete barrier) and 21% involved an oncoming truck. When a motorcycle was driven as the travel mode for suicide, 40% of the collisions involved a fixed object, 47% involved an oncoming truck, and 13% involved an oncoming car/van. Of the pedestrians who used an oncoming vehicle for suicide, 58% collided with a truck and 23% collided with a car/van.

**Table 2:** Proportion of travel modes used and collision counterparts for different types of road vehicle collision as a suicide method between 2001 and 2017 in Australia.

	Fixed object	Car/van	Truck	Bus	Train	Tram	Unknown
Driver deaths by suicide^a^							
Car/van (N = 342)	74%	2%	21%	–	2%	<1%	<1%
Motorcycle^b^ (N = 15)	40%	13%	47%	–	–	–	–
Pedestrian deaths by suicide (N = 235)	1%^a^	23%	58%	6%	–	1%	11%

Not tabulated are collisions involving a bicycle into an oncoming truck, a mobility scooter into an oncoming truck, an unknown vehicle into a fixed object, and an unknown vehicle into an oncoming truck. These collisions accounted for <5% of total drivers who died by suicide.

^a^Includes jumping out of a moving vehicle.

^b^<5% of total drivers who died by suicide.

### Characteristics of individuals who died by suicide involving road vehicle collision and other methods

Table 3 shows that the individual characteristics associated with the likelihood of dying by vehicle-collision suicide were younger age, non-Indigenous origin, and country of birth. Individuals who were younger than 25 years old had more than five times the odds of dying by vehicle-collision suicide compared with those who were 65 years and older (OR 5.27), those aged 25–34 years and 35–44 years had more than triple the odds (OR 3.76 and 3.50, respectively), and those aged 45–54 years had more than double the odds (OR 2.72). Non-Indigenous individuals had triple the odds of dying by vehicle-collision suicide compared with Indigenous individuals (OR 3.36). Individuals born overseas had greater odds of dying by vehicle-collision suicide compared with individuals born in Australia (OR 1.40).

**Table 3:** Descriptive, univariate, and multivariate results for individual characteristics of those who died by suicide involving road vehicle collision and other methods between 2001 and 2017 in Australia.

	Road vehicle collision (N = 640)		Other methods (N = 41,890)		Total	Unadjusted			Adjusted		
	N	%	N	%		OR	95% CI	p-value	OR	95% CI	p-value
Sex								0.3017			0.3156
Male	502	78.44	32129	76.70	32631	1.11	0.91–1.34		1.15	0.88–1.50	
Female^a^	138	21.56	9761	23.30	9899	1.00			1.00		
Age (years)								<0.0001			<0.0001
<25	142	22.22	5692	13.59	5834	3.72	2.62–5.28		5.27	3.05–9.10	
25–34	144	22.54	8135	19.43	8279	2.64	1.86–3.74		3.76	2.20–6.44	
35–44	144	22.54	8863	21.16	9007	2.42	1.71–3.43		3.50	2.08–5.89	
45–54	117	18.31	7917	18.90	8034	2.20	1.54–3.15		2.72	1.60–4.61	
55–64	51	7.98	5155	12.31	5206	1.48	0.98–2.23		1.76	0.99–3.12	
65+^a^	41	6.42	6116	14.60	6157	1.00			1.00		
Unknown	1	–	12	–							
Marital status								0.0041			0.3374
Married/de facto^a^	186	29.06	14357	34.27	14543	1.00			1.00		
Never married	195	30.47	11387	27.18	11582	1.32	1.08–1.62		0.81	0.61–1.08	
Divorced/separated/widowed	116	18.13	9527	22.74	9643	0.94	0.74–1.19		0.96	0.73–1.28	
Unknown	143	22.34	6619	15.80	6762						
Employment status								0.0395			0.7061
Employed	237	37.03	16108	38.45	16345	1.32	1.06–1.64		0.88	0.65–1.19	
Unemployed	124	19.38	9620	22.96	9744	1.16	0.90–1.48		0.91	0.66–1.25	
Not in labor force^a^	130	20.31	11654	27.82	11784	1.00			1.00		
Unknown	149	23.28	4508	10.76	4657						
Indigenous origin								0.0083			0.0005
Indigenous^a^	14	2.19	1972	4.71	1986	1.00			1.00		
Non-Indigenous	490	76.56	33665	80.37	34155	2.05	1.20–3.49		3.36	1.71–6.62	
Unknown	136	21.25	6253	14.93	6389						
Country of birth								<0.0001			0.0060
Australia^a^	386	60.31	23555	56.23	23941	1.00			1.00		
Overseas	253	39.53	6939	16.56	7192	2.22	1.89–2.61		1.40	1.10–1.79	
Unknown	1	0.16	11396	27.20	11397						

OR, odds ratio; CI, confidence interval.

^a^ Reference category

## Discussion

The current study examined the annual suicide rate involving road vehicle collision and other methods for the period of 2001–2017, the proportion of different types of vehicle collisions, and individual characteristics associated with the likelihood of dying by vehicle-collision suicide compared with other suicide methods. We observed an overall increase in the national suicide rate involving road vehicle collision over the study period, with the rate being significantly greater in 2012, 2015, and 2017 than in 2001. The rate consistently increased between 2005 and 2008 (an aggregate of the upward trend observed in Queensland, Victoria, Western Australia, and Tasmania in the same period). In contrast, we observed an overall decline in the national suicide rate involving other methods throughout the same time period, with the rate being significantly lower in each year since 2003 compared with 2001.

It is worth considering the factors that may explain why deaths by suicide involving road vehicle collision have increased in the face of a decline in deaths by suicide involving other methods. Some substitution may be occurring, with an increasing availability of cars (road vehicle use consistency increased between 2001 and 2017 in Australia [19–21]). In addition, suicide prevention efforts that may have reduced deaths by suicide involving other methods (e.g., the annual incidence of suicide deaths by motor vehicle exhaust gas has decreased by 57% between 2001 and 2006 [23]) may have inadvertently led to an increase in deaths by vehicle-collision suicide. However, these explanations are at best only likely to partially explain the diverging trends observed in the current study. Deaths by vehicle-collision suicide continue to account for a small proportion of all deaths by suicide, these relatively small numbers make it difficult to definitely determine the cause of their changing patterns.

We found that most suicide events involving road vehicle collision were by drivers, which is consistent with studies conducted in Finland [5], Scotland [6], Sweden [8], and Switzerland [7]. Of the drivers who died by suicide, 72% were in single-vehicle collisions, which is greater than 55% [10] and 59% [11] reported in earlier Australian studies. Car/van was the predominant mode of travel used and motorcycles were occasionally used, with the most common collision counterpart being a tree. For the remaining drivers who died by suicide, 27% were in multiple-vehicle collisions. Of these drivers, 82% collided with an oncoming truck. This estimate is consistent with an earlier Australian study [10] and considerably greater than the 28% reported in another Australian study [11]. However, these earlier studies used comparatively smaller sample sizes than the current study (N = 10 and 52 vs. N = 365). In addition, we found that more than one-third of suicide events involving road vehicle collision were by pedestrians, with the most common collision counterpart being an oncoming truck. This estimate is consistent with an earlier study in Scotland which also used a smaller sample size (N = 17).

We identified individual characteristics associated with likelihood of dying by vehicle-collision suicide in comparison to other suicide methods. Individuals younger than 25 years old had the highest likelihood of dying by vehicle-collision suicide among all age groups. Specifically, they were five times more likely to die by vehicle-collision suicide than individuals aged 65 years and older. Those aged 25–44 years and 45–54 years were more than three times and two times more likely to die by vehicle-collision suicide, respectively, than individuals aged 65 years and older. Males were more likely to die by vehicle-collision suicide than females. This supports earlier Australian studies that reported drivers who died by vehicle-collision suicide are often by younger men [10, 11, 13, 14], which is a finding that is also observed in other countries [1, 3, 5, 7, 14–18]. Our findings also indicated that individuals who were non-Indigenous were three times more likely to die by vehicle-collision suicide than those who were Indigenous, and individuals born overseas were more likely to die by vehicle-collision suicide than those born in Australia. Unemployed individuals were not more likely to die by vehicle-collision suicide than those employed and not in the labor force, in contrast to an earlier Australian study [11].

Different types of initiatives are available to reduce suicide events involving road vehicle collision. First, general road safety measures for preventing unintentional road deaths are likely to have suicide preventive benefits. For example, setting reduced speed limits may reduce pedestrian deaths by vehicle-collision suicide (pedestrian fatality risk rapidly increases with any small increase in vehicle-pedestrian impact speed between 30–70km/hr [24]). Installing cable barriers and guardrails on both sides of a road and between opposite direction lanes may also reduce driver deaths by suicide in single-vehicle collisions and multiple-vehicle collisions, respectively (cf. concrete barriers and ditches which are associated with a relatively higher risk of injury in road vehicle collisions [25]). However, Australia has a vast road network and road vehicles are widely accessible. Initiatives that involve the installation of infrastructure (e.g., signs, road safety barriers, crisis telephones, and CCTV) may face feasibility and cost effectiveness issues, and would likely be challenging to implement especially over a large area. Technological innovations in vehicles that improve general road safety may also prevent suicide events involving road vehicle collision. An earlier Australian study found that most drivers who died by vehicle-collision suicide had consumed alcohol prior [11]. Furthermore, the relationship between alcohol use and increased predisposition toward suicidal behavior is well known [26–28]. Alcohol interlocks in vehicles can have a role in restricting vehicle access to an at-risk individual who is under the influence of alcohol and intend to self-harm. ‘Smart’ interlocks can also be adopted to prevent an at-risk driver from dying by suicide via remote lockdown of a vehicle to secure the individual inside long enough for assistance to arrive. In addition, vehicle systems can monitor driving behaviors that correspond to those of drivers who died by suicide in vehicle collisions (e.g., abrupt sharp steering, excessive speeding, not wearing a seat belt) to trigger for reduction of speed and warning signals for oncoming vehicles.

Second, road vehicle collision as a suicide method should be the focus of targeted interventions. For example, refining media guidelines to specifically advise on responsible reporting of road vehicle collision fatalities [29] may reduce the risk of so-called “copycat” behavior [30, 31]. For social media (e.g., Facebook, Twitter), amending platform guidelines and releasing platform updates can help to reduce the spread of harmful content, promote help-seeking behavior, and direct at-risk users to crisis support services (e.g., Lifeline). Based on our findings, such efforts might preferentially target some groups (e.g., males younger than 25 years old, individuals who were non-Indigenous and born overseas). Our findings also indicate that oncoming trucks were involved in most pedestrian deaths by suicide and driver deaths by suicide in multiple-vehicle collisions. Therefore, enhancing national assessment practices for heavy vehicle licensing (e.g., vehicle-collision suicide awareness training, defensive driving training, simulations of suicidal behavior in hazard perception testing) may reduce the risk of death from vehicle collision suicide by pedestrians and drivers. The installation of signs to display positive messaging and information about help services, crisis telephone support services to increase opportunities for help-seeking, and blue light-emitting diode lamps (which has been shown to reduce suicide deaths at railway stations with no substitution at neighbouring stations [32]) may also reduce suicides by vehicle collision, especially at areas where such suicide events are frequent. Last, general primary prevention of suicide, especially measures targeting adolescents (i.e., school-based programs), are likely to also reduce suicides by road vehicle collision. These involve problem-solving training, basic mental health education, encouragement of help-seeking behavior, gatekeeper training, and public awareness campaigns that empower individuals to talk about suicide [33, 34].

Our study examined the most representative dataset on suicide deaths involving road vehicle collision in Australia to date, compiling coronial data from each Australia state and territory across a 17-year period (2001–2017). To our knowledge, this is the most comprehensive study on road vehicle collision as a suicide method in Australia, and the first to report its trend over time. Our study is also the first to examine pedestrians who died by suicide involving road vehicle collision in Australia.

Nevertheless, our conclusions are limited by the likely underestimation of the true number of drivers who died by suicide. Underestimation of drivers who died by suicide is widely acknowledged [5–7, 9, 14, 15, 18], and is due to a range of difficulties faced by coroners in making an explicit determination of intent. Sufficient evidence on critical background and contextual information about the deceased may at times be unavailable [2, 10, 11, 18, 35–39]. This includes indicators of psychological condition (e.g., history of suicidal behavior and ideation), emotional state (e.g., a suicide note), and driver behavior prior to the collision (e.g., eyewitness accounts). A suicide verdict requires a standard of proof with a high degree of certainty, which naturally vary among coroners even given the same key information [39–45]. The result is potential misclassification of drivers who genuinely died by suicide as accidents [9, 18, 46, 47] and due to undetermined causes (which remain as open verdicts and are not in any official statistics). Underestimation of the true number of drivers who died by suicide has been acknowledged in studies conducted in Australia [11] and other countries [5, 46, 47], and could explain the large discrepancy in vehicle-collision suicide rates among other countries in the past five decades (between 1.6% and 32% of all road fatalities [1–3, 5–8, 15, 16, 48–50]).

Future research should focus on identifying immediate precursors to suicide events involving road vehicle collision. These include blood alcohol level (most drivers who died by suicide in Queensland, Australia, were intoxicated prior to the collision, more so than other methods of suicide combined [11]), presence of illicit substances, existing mental health issues, and exposure to recent adverse life events (e.g., legal, financial, and marital problems). Subsequent studies should also focus on identifying specific areas where deaths by suicide involving road vehicle collision are frequent. This would enable the implementation of interventions at problematic sites (e.g., signs, road safety barriers, blue light-emitting diode lamps) that may reduce deaths by vehicle-collision suicide.

## Conclusions

This research provides a longitudinal profile of road vehicle collision suicide in Australia. Our conclusions are: (i) the vehicle-collision suicide rate increased over time while the suicide rate by other methods decreased; (ii) vehicle-collision suicide events were predominantly by drivers in single-vehicle collisions and a substantial minority by pedestrians; (iii), individuals who die by vehicle-collision suicide are more likely to be young males, non-Indigenous, and born overseas when compared to individuals who die by other methods. Our findings inform future research directions and prevention initiatives for road vehicle collision suicide in Australia.
